# Energy drinks and psychotic symptoms. A case report

**DOI:** 10.1192/j.eurpsy.2025.1063

**Published:** 2025-08-26

**Authors:** D. Mancho Gragera, C. A. Díez de los Ríos, M. S., S. O. Molina

**Affiliations:** 1 Psiquiatría y Salud Mental, Hospital Universitario Ramón y Cajal; 2 Psiquiatría y Salud Mental, Hospital Universitario Infanta Leonor, Madrid, Spain

## Abstract

**Introduction:**

This is a 26-year-old male patient, diagnosed with bipolar disorder, admitted to a medium-stay unit for two months due to lack of awareness of the disease, after several admissions to the acute unit for manic decompensation with psychotic symptoms after abandoning psychopharmacological treatment and cannabis use.

The patient, who was stable since the beginning of the medium-stay admission after being referred directly from an acute unit, began to show strange behavior that became more pronounced over the days.

**Objectives:**

To study the relationship between energy drinks and the development of psychotic symptoms in the existing literature, based on a clinical case.

**Methods:**

Description of the clinical case.

Bibliographic review related to the topic.

**Results:**

The difficulty in this case was to find out the cause of the psychotic symptoms that the patient was presenting, since he was taking the psychopharmacological treatment well and the urine tests that were carried out every time he returned from the street were negative. He had not had any stressful events either. On one of the outings that he made, an assistant saw that he was drinking a can of Monster. When asked, the patient commented that he usually drank between one and two cans a day. Olanzapine 10 mg (0-0-1) and clonazepam 2 mg (1-0-1) were added to the patient that he was taking. In addition, his outing permits will be withdrawn until clinical stabilization is achieved.

**Conclusions:**

In the acute appearance of psychotic symptoms in patients with a previous history, possible causes must always be investigated, the most frequent being the following: abandonment of psychopharmacological treatment, consumption of toxic substances and stressful events. Although it is true that there are not many studies that support this, cases have been reported in which energy drinks, due to their high caffeine content, could also be one of the triggers.

**Disclosure of Interest:**

None Declared

